# Emergency Laparoscopic Removal of an Ingested Fishbone Perforating the Stomach After Endoscopy: A Case Report

**DOI:** 10.7759/cureus.81060

**Published:** 2025-03-23

**Authors:** Norio Tomono, Naohisa Niiro, Yasuhiko Fujita, Teruyoshi Amagai

**Affiliations:** 1 Surgery, Tokunoshima Tokushukai General Hospital, Kagoshima, JPN; 2 Internal Medicine, Amagi Yuino-Sato Medical Center, Kagoshima, JPN; 3 Obstetrics and Gynaecology, Tokunoshima Tokushukai General Hospital, Kagoshima, JPN; 4 Radiology, Kagoshima Tokushukai General Hospital, Kagoshima, JPN; 5 Radiology, Tokunoshima Tokushukai General Hospital, Kagoshima, JPN; 6 Clinical Engineering, Faculty of Health Care Sciences, Jikei University of Health Care Sciences, Osaka, JPN; 7 Internal Medicine, Tokunoshima Tokushukai General Hospital, Kagoshima, JPN

**Keywords:** endoscopy, fishbone, gastric perforation, laparoscopy, multi-detector computed tomography (mdct)

## Abstract

A 70-year-old female who presented with epigastric pain of two days' duration was reported. Her multi-detector computed tomography (MDCT) images showed peri-gastric fat stranding on MDCT, corresponding to the extra-gastric wall inflammation due to gastric perforation. Laparoscopic surgery was successfully performed to remove the extra-gastric foreign body and repair the perforation with the lesser omentum. To the best of our knowledge, the literature review showed that this is the 10th case of gastric perforation due to a foreign body treated by laparoscopy. The fat stranding is considered indicative of gastric perforation, and laparoscopy must be prepared when gastroscopy fails to remove and save the gastric perforation.

## Introduction

For foreign bodies in the stomach, the diagnosis is made by measuring the size and confirming the location using abdominal computed tomography (CT), followed by emergency endoscopy. If the foreign body cannot be removed by emergency endoscopy, a case-by-case approach, depending on the size and type of the foreign body, is suggested (weak recommendation and low-quality evidence) [1]. Ingestion of fish bones is a common occurrence, and diagnosis by multi-detector computed tomography (MDCT) is extremely important. However, there are few reports of perigastric fat stranding in addition to intraperitoneal free air as an MDCT finding for perforation of the gastric wall, the thickest part of the digestive tract wall. Here, we report a case in which fat stranding around the stomach was observed on MDCT of a fishbone, and the site of gastric perforation was such that endoscopic removal of the fishbone was not possible, requiring treatment with laparoscopic surgery to remove the fishbone and then fill the perforated site with the lesser omentum.

## Case presentation

A 70-year-old female presented with epigastric pain of two days' duration. Two days ago, the patient swallowed a fishbone after eating boiled sea bream. In the evening of the same day, epigastric pain appeared. The symptoms persisted the next day, so the patient went to a local doctor. The MDCT images revealed a high-density foreign body in the antrum of the stomach, and the patient was referred to our hospital. The patient's medical history included reflux esophagitis, depression, and a history of appendectomy for appendicitis in her 20s. She was not taking any medication. Her last meal and drink had been six hours prior to the presentation. The patient's social history included nonsmoking and occasional drinking. On physical examination, her consciousness was clear, body temperature 35.2°C, blood pressure 132/77 mmHg, heart rate 77/min, respiratory rate 16/min, and SpO_2_ 98% (on room air). Her physical findings revealed a flat and soft abdomen, epigastric tenderness, and muscular rigidity. The laboratory data from the venous sample on arrival are shown in Table 1.

**Table 1:** Laboratory panel on the arrival at our hospital Abbreviations: Alb: albumin; ALT: alanine aminotransferase; AST: aspartate aminotransferase; BE: base excess; BNP: brain natriuretic peptide; BUN: blood urea nitrogen; CBC: complete blood count; CRP: C-reactive protein; eGFR: estimated glomerular rate; Hb: hemoglobin; Ht: hematocrit; MCH: mean corpuscular hemoglobin; MCV: mean corpuscular volume; PCO_2_: carbon dioxide partial pressure; Plt: platelet; PO_2_: oxygen partial pressure; RBC: red blood cell; T-bil: total bilirubin; WBC: white blood cell

Category	Item	Data	Reference	Unit
Venous gas	pH	7.369	7.35～7.45	-
	PCO_2_	41.6	-	torr
	PO_2_	37.1	-	torr
	BE	-1.8	-	mEq/L
CBC	WBC	6080	3300～8600	count/μL
	RBC	367	386～492	count/μL
	Hb	11.1	35.1～44.4	g/dL
	Ht	33.8	83.6～98.2	%
	MCV	92.1	27.5～33.2	fL
	MCH	32.8	31.7～35.3	pg
	Plt	18.8	15.8～34.8	x10^4 ^count/μL
Biochemistry	AST	22	13～30	U/L
	ALT	18	7～23	U/L
	Alb	3.5	4.1～5.1	g/dL
	T-Bil	1.25	0.4～1.5	mg/dL
	BUN	7.6	8.0～20.0	mg/dL
	Creatinine	0.7	0.46～0.79	mg/dL
	Na	141.7	138～145	mEq/L
	K	4	3.6～4.8	mEq/L
	Cl	101.8	101～110	mEq/L
	CRP	10.89	0.00～0.14	mg/dL
	eGFR	84.7	90～	mL/min/1.73 m^2^
	BNP	19.5	0.0～18.4	pg/mL
	HbA1c	5.3	4.9～6.0	%

**Figure 1:**
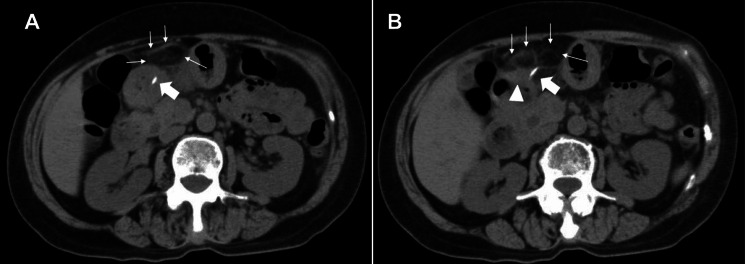
Multidetector computed tomography (MDCT) images of a fish bone-like structure and extra-gastric fat stranding showing inflammation of the stomach wall The marks included in this figure are as follows: Thick Arrow: a linear fish-bone-like abnormality extending from the anterior wall of the gastric antrum (A) to the outside of the gastric wall (B), representing a high-density structure; Thin Arrow (A, B): thickening of the stomach wall in the antrum; Triangle (B): extra-gastric fat stranding.

**Figure 2:**
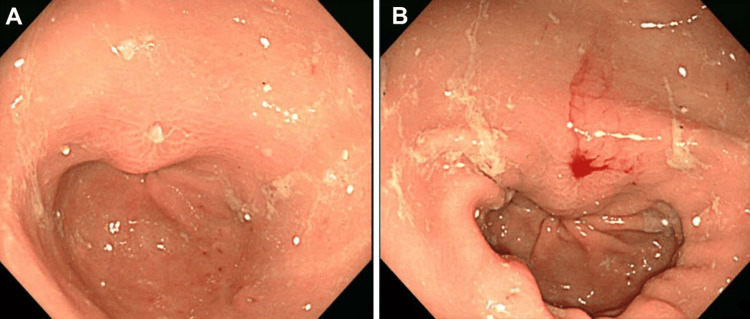
Gastroscopic images This picture reveals the pin-hole appearance of the ulceration (A), and no foreign body was detected in the gastric mucosal layer (B).

With the exception of a higher C-reactive protein (CRP) than normal, a marker of inflammatory response, all blood count and biochemistry data were within normal limits. The CT images obtained in our hospital showed a 3 cm long, highly attenuated linear shadow on the gastric antrum (Figure 1). Under the diagnosis of an intra-gastric ingested fishbone perforating into the peritoneum, a gastroscopy was performed for observation and subsequent removal of this foreign body. The gastroscopy revealed gastric mucosal edema and central ulceration. The ulcerated area was thought to have an embedded fishbone with surrounding inflammation (Figure 2).

**Figure 3:**
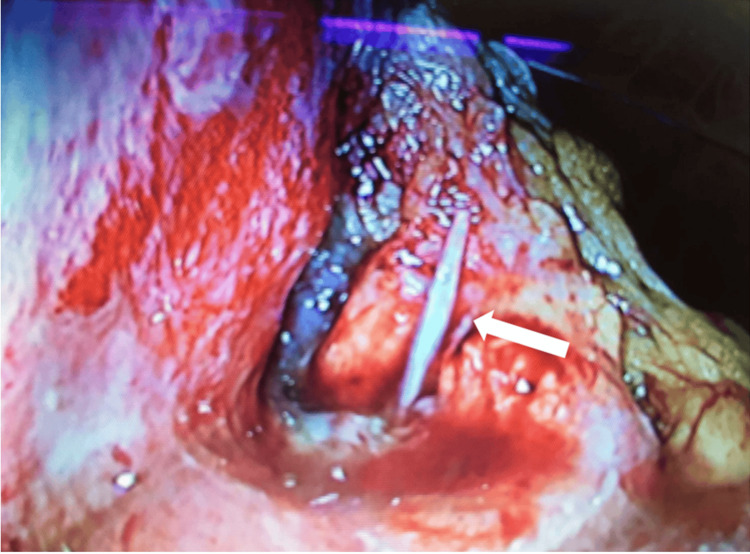
Appearance of stomach penetrated foreign body seen through laparoscopy A white foreign body (arrow) is visible outside the stomach's outer layer. There is also swelling, and yellow-spotted abscesses were seen around the foreign body.

**Figure 4:**
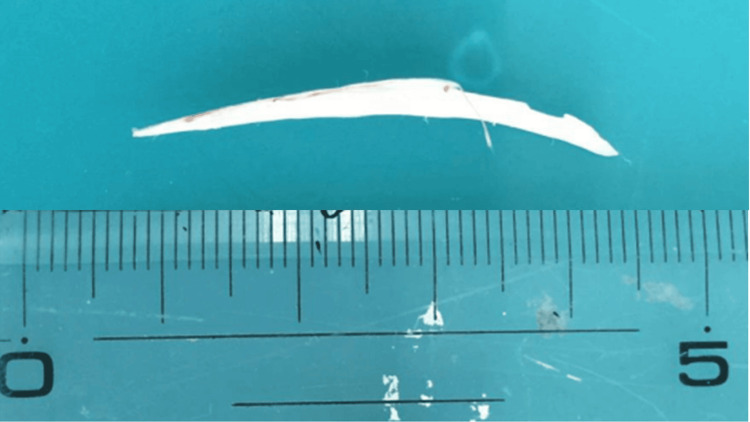
Appearance of removed fishbone The foreign body removed from the stomach through laparoscopy was a fish bone, 37 millimeters long.

Although we approached the area with forceps, she was unable to extract the fishbone. Therefore, urgent laparoscopic surgery was performed under general anesthesia due to her peritoneal signs after endoscopy. Three ports were placed in the umbilicus, right lower quadrant, and supra-umbilical midline. The white foreign body was found to have penetrated through the lesser curvature of the stomach and was easily removed with forceps (Figures 3-4).

The perforated stomach was then packed through the lesser omentum with two sutures. The drainage tube was placed in the liver bed to monitor for leakage. On postoperative day (POD) 5, the upper gastrointestinal (GI) contrast study showed no leakage from the stomach, and the drainage tube was removed the next day. Her post-laparoscopic course was uneventful, and she was discharged home on POD 7 without medication.

## Discussion

Laparoscopic surgery for gastric perforation from foreign body ingestion

Surgical management is indicated in cases of peritoneal signs secondary to perforation, abscess formation, blood vessel penetration, severe inflammation, or bleeding [2]. We searched the literature on gastric perforation caused by the ingestion of foreign bodies, screening through titles and abstracts using the keywords “fishbone,” “perforation,” “stomach,” and “laparoscopy” via PubMed on February 12, 2025. As a result of the search, 17 cases from 14 studies fully met the inclusion criteria of the above search terms, out of 102 cases. The clinical profiles, MDCT findings, and survival outcomes are shown for all cases, including ours. The cases of gastric perforation by ingested foreign bodies were treated with laparoscopic surgery [3-10] and without laparoscopic surgery [11-16]. The age, preoperative time, and MDCT findings of the collected cases varied widely. We could not identify the tendencies in cases of gastric perforation caused by ingested foreign bodies (Tables 2-3).

**Table 2:** Case list of foreign body-related gastric perforation treated by laparoscopic surgery Abbreviations: GS: gastroscopy; LS: laparoscopic surgery; GI: gastrointestinal; LOS: postoperative length of stay in hospital; NA: not available; size: maximal length of the foreign body; CT: computed tomography

Case	Age	Sex	Complains	Visit timing	Foreign body	Size	CT findings	GS	LS	LOS days	Outcome	Year	Reference
1	80	Male	Epigastric pain	1 day	Fishbone	25	Posterior wall of antrum	+	+	7	Survived	2018	[3]
2	23	Female	Epigastric pain, retrosternal burn	NA	Sewing needle	15	A foreign body presenting starts from the posterior side of the stomach and reaching the head and body of the pancreas	-	+	6	Survived	2018	[3]
3	63	Male	Epigastric pain	1 month	Fishbone	25	Linear, hyperdense, foreign body perforating the lesser gastric curvature	+	+	7	Survived	2019	[4]
4	67	Male	Epigastric pain	3 months	Fishbone	32	Linear, hyperdense, foreign body along the stomach wall and pancreatic neck	+	+	5	Survived	2021	[5]
5	56	Male	Intermittent chest tightness and shortness of breath	7 days	Fishbone	20	Needle-like high-density shadow in the liver segment Ⅳ	-	+	5	Survived	2022	[6]
6	26	Female	Abdominal pain	2 months	Sewing needle	50	A needle outside the GI tract and on the pancreas surface	-	+	2	Survived	2022	[7]
7	43	Male	Fever, headache	8 days	Fishbone	30	liver abscess (*Streptococcus anginosus*, *Actinomyces odontolyticus*)	+	+	7	Survived	2023	[8]
8	36	Female	Epigastric pain	3 days	Metalic wire	30	Posterior lesser curvature	+	+	10	Survived	2023	[9]
9	53	Female	Epigastric pain to back	2 weeks	Toothpick	NA	A thin linear radiopaque lesion with an adjacent low attenuating lesion in S3 of the liver	-	+	5	Survived	2023	[10]
10	70	Female	Epigastric pain	2 days	Fishbone	37	Perigastric fat stranding and thickening of anterior wall of the antrum	+	+	7	Survived	2025	Present case

**Table 3:** Case list of foreign body-related gastric perforation treated by gastroscopy without laparoscopic surgery Abbreviations: GS: gastroscopy; LS: laparoscopic surgery; LOS: postoperative length of stay in hospital; NA: not available; size: maximal length of the foreign body; CT: computed tomography

Case	Age	Sex	Complains	Visit timing	Foreign body	Size	CT findings	GS	LS	LOS	Outcome	Year	Reference
1	58	Male	Epigastric pain radiating to the back	2 months	Fishbone	40	Fat stranding at distal stomach, duodenum	+	-	7	Survived	2019	[11]
2	80	Female	Abdominal pain	2 days	Chicken bone	40	Perforating the full thickness of the gastric wall	+	-	2	Survived	2019	[11]
3	78	Female	Abdominal pain	2 days	Fishbone	5	A linear high-intensity structure penetrating the anterior wall of the antrum	+	-	NA	Survived	2021	[12]
4	69	Female	Epigastric pain	4 days	Fishbone	5	A linear high-intensity structure penetrating the posterior wall of the antrum	-	-	NA	Survived	2021	[12]
5	43	Female	Abdominal pain	3 weeks	Ink pen	NA	A linear foreign body in the distal gastric antrum penetrating the inferior wall and extending into the adjacent peritoneal fat	+	-	NA	Survived	2021	[13]
6	10	Male	Epigastric pain to back	5 days	Metalic wire	40	A bulky and oedematous pancreas with a 4 × 3.5 cm area of parenchymal necrosis in the distal body	+	-	5	Survived	2023	[14]
7	43	Male	RUQ abdominal pain, diarrhea	2 days	Fishbone	30	Curvilinear hyperdense foreign body extending from the first part of the duodenum	+	-	3	Survived	2024	[15]
8	21	Male	Hematemesis	1 day	Chicken bone	NA	A linear, calcified, and sharp foreign object lodged in the antrum	+	-	3	Survived	2024	[16]

MDCT for identification of gastric perforation

MDCT findings of gastric perforation are as follows: a discontinuity of the bowel wall, the presence of extraluminal air, and indirect CT findings such as bowel wall thickening, abnormal bowel wall enhancement, abscess, and an inflammatory mass adjacent to the bowel [17]. Diseases showing fat stranding in CT findings include appendicitis, cecal diverticulitis, and perforated cecal carcinoma [18]. In addition, it is reported that a high positive predictive value (PPV) of perivisceral fat stranding due to colonic perforation, general distension of upstream loops, and collapse of downstream loops were evident [19].

Recently, the presence of peri-enteric fat stranding, kappa 0.45 (0.23, 0.69), has been added to the above findings [20]. Regarding MDCT findings in 18 case series of gastric perforation (Tables 2-3), peri-enteric fat stranding is reported only in our case. From these results, we would like to emphasize that fat stranding around the GI tract in MDCT findings must be added to the other CT findings.

## Conclusions

A 70-year-old female who presented with epigastric pain of two days' duration was reported. Her MDCT images showed peri-gastric fat stranding on MDCT, corresponding to the extra-gastric wall inflammation due to gastric perforation, which was successfully removed by laparoscopic surgery and repaired with the lesser omentum. To the best of our knowledge, the literature review showed that this is the 10th case of gastric perforation due to a foreign body treated by laparoscopy.
